# Otological Findings Ten Years after Myringotomy with Tympanostomy Tube Insertion

**Published:** 2012

**Authors:** Behrouz Barati, Seyed Mostafa Hashemi, Ali Goljanian Tabrizi

**Affiliations:** 1 *Department of Otorhinolaryngology and Head and Neck Surgery, Shahid Beheshti University of Medical Sciences, Tehran, Iran.*; 2 *Department of Otorhinolaryngology and Head and Neck Surgery, Isfahan University of Medical Sciences, Isfahan, Iran.*

**Keywords:** Myringotomy, Myringosclerosis, Otitis media with effusion, Perforation, Tympanostomy tube

## Abstract

**Introduction:**

To study the long-term complications of tympanostomy tube insertion in young children 10 years after surgery.

**Materials and Methods:**

In September 2011, the medical records of all patients who had undergone myringotomy with tympanostomy tube insertion between February 2000 and March 2001 at the two general hospitals of Isfahan University of Medical Sciences were studied. Of the 98 patients who fulfilled the inclusion criteria, 82 patients agreed to participate and were enrolled in the study. The complications of the operation were evaluated in these patients.

**Results:**

Of the 164 ears that were operated on, myringosclerosis was found in 17.1%, atrophy of the tympanic membrane in 1.2%, permanent perforation of the tympanic membrane in 0.6% and tympanic membrane atelectasis in 0.6%. None of the patients developed cholesteatoma as a complication of tympanostomy tube insertion.

**Conclusion:**

Considering the low risk of serious complications after 10 years, tympanostomy tube insertion is a safe and effective treatment option in the treatment of otitis media with effusion.

## Introduction

Otitis media with effusion (OME) is defined as collection of fluid in the middle ear cleft, in the presence of an intact tympanic membrane and with no evidence of inflammation (1). Between 35% and 70% of pre-school-aged children experience at least one episode of OME (2) and approximately 17% to 41% of children between the ages of 2 and 3 years old suffer from OME (3). The development of OME can either be as a result of Eustachian tube obstruction, or due to acute or chronic otitis media. Plenty of studies have evaluated the use of antibiotics in patients with OME and most of the studies indicate that antibiotics are not effective in treating OME. Other studies, however, have shown good results in children with OME following myringotomy with insertion of a tympanostomy tube (1).

Indications for performing a myringotomy with tympanostomy tube insertion in OME include:

1. OME lasting 4 months or longer with persistent hearing loss or other signs and symptoms;

2. Recurrent or persistent OME associated with increased risk of developmental problems regardless of hearing status;

3. OME and structural damage to the tympanic membrane or middle ear (4).

Myringotomy with tympanostomy tube insertion can create complications such as permanent perforation, atrophy of the tympanic membrane, retraction and atelectasis of the tympanic membrane and cholesteatoma.

Since this operation is one of the more common operations performed under general anesthesia in children (5), and there are only a few studies regarding its long-term complications, we decided to study the long-term complications of the procedure and compare them with similar studies performed elsewhere.

## Materials and Methods

This study included all the patients who had undergone myringotomy with tympanostomy tube insertion at Al-Zahra General Hospital and Kashani General Hospital in Isfahan between February 2000 and March 2001. In September 2011, the medical records of more than 500 patients were studied and those who fulfilled the following inclusion criteria were recruited:

1. OME resistant to appropriate antibiotic therapy that required treatment with myringotomy and tympanostomy tube insertion.

2. Insertion of bilateral tympanostomy tubes.

3. Placement of the grommet style of tube,

4. Aged between 2 and 4 years old at the time of surgery.

*Exclusion criteria included:*

1. Craniofacial anomalies such as cleft lip or palate.

2. Placement of special tympanostomy tube styles such as Paparella tubes, T-tubes or Collar Bobbin tubes.

3. Patients requiring repeat tympanostomy tube insertion.

Of the 98 patients who fulfilled the inclusion criteria, 82 patients agreed to participate and were enrolled in the study. An ear examination was performed with a microscope and the complications of the operation were studied and analyzed in the included patients. Parental consent was obtained and all the examinations were performed free of charge.

## Results

In this survey, 82 patients who had undergone bilateral myringotomy with tympanostomy tube insertion for OME 10 years prior to the study were examined and the statistical findings were analyzed. Out of 82 patients, 48 (58.5%) were male and 34 (41.5%) were female. After 10 years none of the patients still retained a tympanostomy tube or had ongoing OME. Post-operative myringosclerosis was found in 25 (31.5%) patients, which was unilateral in 22 (26.8%) and bilateral in 3 (3.6%) patients. Of the 164 ears that had been operated on, 28 (17.1%) had myringosclerosis. Unilateral post-operative myringosclerosis was present in the right ear in 13 (7.9%) patients and in the left ear in 9 (5.5%) patients (Table 1).

**Table 1:** Prevalence of myringosclerosis as a complication of myringotomy with tympanostomy tube insertion

	Number of ears (N = 164)	Percentage (%)
Unilateral Myringosclerosis in the Right Ear	13	7.9
Unilateral Myringosclerosis in the Left Ear	9	5.5
Bilateral Myringosclerosis	6	3.7
No Myringosclerosis	136	82.9

**Fig 1:**
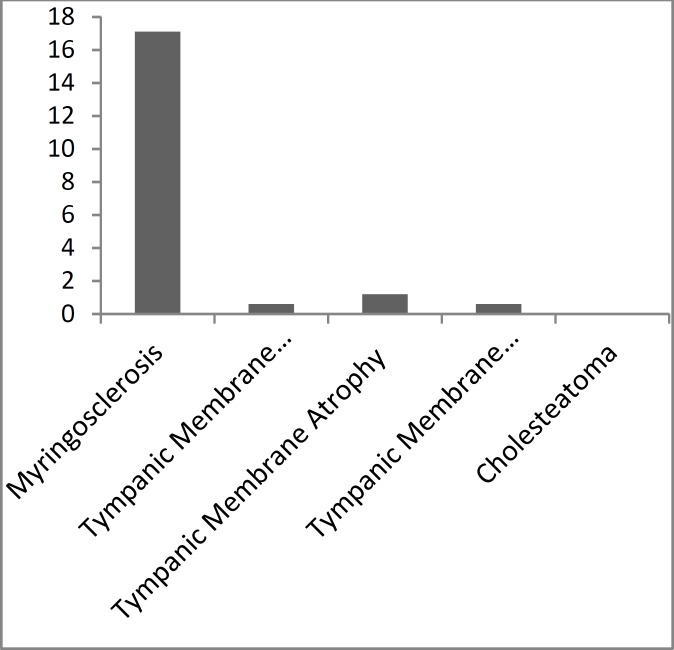
Prevalence (%) of complications 10 years after myringotomy with tympanostomy tube insertion

Perforation of the tympanic membrane was found in 1 (0.6%) ear, in 2 (1.2%) ears there was atrophy of the tympanic membrane, and in 1 (0.6%) ear there was atelectasis of the tympanic membrane. None of the patients had cholesteatoma (Fig. 1).

## Discussion

Mandel and colleagues studied 109 children with OME that was resistant to treatment with antibiotics and showed that myringotomy with tympanostomy tube insertion is associated with longer otitis media-free periods and better hearing compared with myringotomy alone and non-surgical approaches (5). Similarly, a study by Rosenfeld and colleagues revealed that myringotomy with tympanostomy tube insertion improves the quality of life in children with OME (6). Bluestone and colleagues further concluded that simple myringotomy either by laser or cold knife is not the method of choice in preventing the recurrence of otitis media (7).

Myringosclerosis is the deposition of hyaline and calcium in the collagen layer of the tympanic membrane. It often occurs in patients who have undergone tympanostomy tube insertion (8,9). Mattsson and colleagues proposed that the pathogenesis of myringosclerosis might be due to increased production of oxygen-derived free radicals as an outcome of hyperoxic conditions leading to the accumulation and aggregation of sclerotic deposits (10). Trauma is a major factor in the development of myringosclerosis, and is most commonly associated with myringotomy and tympanostomy tube insertion. Intratympanic hemorrhage is also implicated in the pathogenesis of myringosclerosis and tympanosclerosis (8, 11). The rate of occurrence of myringosclerosis after myringotomy with tympanostomy tube insertion was reported to be 48% by Bonding and Tos (12), 42% by Brown and colleagues (13), and 52% by Pichichero and colleagues (14). Kay and colleagues reported a 32% incidence of myringosclerosis in their meta-analysis (15). Myringosclerosis was the most common complication of tympanostomy tube insertion in our study and we found a 17.1% incidence rate of myringosclerosis in ears that had been operated on.

Tube insertion may also lead to atrophy of the tympanic membrane (16, 17). Johnston and colleagues reported atrophy of the tympanic membrane as the most common complication following tube insertion as it occurred in 74.7% of ears with tubes (18). Maw and Bawden (17) and Yaman and colleagues (19) found tympanic membrane atrophy in 22% and 23.5% of ears with tubes, respectively. In comparison, ears with otitis media but no tubes develop focal atrophy in 14% of cases (15). In the study by Brown and colleagues, 13% of patients had tympanic membrane atrophy (13). We found atrophy in 1.2% of ears that had been operated on.

Tympanic membrane retraction and atelectasis was found in 1.6% of patients in a study by McLelland (20). In contrast, Valtonen and colleagues reported a point-prevalence of 16.7% for tympanic membrane retraction in their 5-year follow-up study (21). Daly and colleagues found atelectasis in 38% of ears with tubes (22). Only 0.6% of ears that were operated on developed this complication in our study.

Permanent perforation is one of the most serious complications following tympanostomy tube insertion, because it requires additional intervention. Bingham and colleagues suggested that 1 year should be the cutoff time for a perforation to be considered as a permanent perforation (23). Wilson suggested that the cause of a tympanic membrane perforation secondary to tympanostomy tube insertion might be due to the growth of squamous epithelium onto the undersurface of the tympanic membrane (24). Perforation of the tympanic membrane was observed in 1% of patients in a study conducted by Curley (25). Perforation was reported in 13% to 18% of patients by Matt and colleagues (26) and in 3.1% of patients by McLelland (20). Other investigators have reported rates of perforation of the tympanic membrane of between 1.1% and 9% following tympanostomy tube insertion (27, 28). We found an incidence rate of 0.6% for tympanic membrane perforation in our study.

Cholesteatoma was found in 0.1% of cases in McLelland’s study (20). Kay and colleagues’ meta-analysis revealed a mean prevalence of cholesteatoma of 0.8% (15). Valtonen and colleagues found no cholesteatoma in their patients (21). We also did not encounter cholesteatoma as a complication of myringotomy with tympanostomy tube insertion.

## Conclusion

The incidence of complications following myringotomy with tympanostomy tube insertion was lower in our study compared with similar studies. This difference could be due to racial factors, a longer follow-up period or the style of tympanostomy tube used in the operations. From our study, it can be concluded that myringotomy with tympanostomy tube insertion is not associated with serious long-term complications, and should be strongly considered as a treatment option for OME that is resistant to treatment with antibiotics.
